# Oral health behaviors for young low-income urban children during the COVID-19 pandemic: a mixed methods analysis

**DOI:** 10.21203/rs.3.rs-2956733/v1

**Published:** 2023-05-25

**Authors:** Molly A Martin, Vyshiali Sundararajan, Nadia Ochoa, John Dziak, Michael Berbaum, Helen H Lee, David M Avenetti, Tong Zhang, Anna Sandoval, Javier Torres, Andy Wu

**Affiliations:** University of Illinois Chicago; University of Illinois Chicago; University of Illinois Chicago; University of Illinois Chicago; University of Illinois Chicago; University of Illinois Chicago; University of Illinois Chicago; University of Illinois Chicago; University of Illinois Chicago; University of Illinois Chicago; University of Illinois Chicago

**Keywords:** COVID-19, health behaviors, oral health, toothbrushing, diet habits

## Abstract

This research assessed oral health behaviors changes in urban families with young children during the stay-at-home period of the COVID-19 pandemic. Survey data on oral health behaviors were collected in homes at three points over one year before COVID-19, and then via phone during COVID-19. Multivariate logistic regression was used to model tooth brushing frequency. A subset of parents completed in-depth interviews via video/phone that expanded on oral health and COVID-19. Key informant interviews via video/phone were also conducted with leadership from 20 clinics and social service agencies. Interview data were transcribed and coded, and themes were extracted. COVID-19 data collection went from Nov 2020 – August 2021. Of the 387 parents invited, 254 completed surveys in English or Spanish (65.6%) during COVID-19. Fifteen key informant (25 participants) and 21 parent interviews were conducted. The mean child age was approximately 4.3 years. Children identified as mainly Hispanic (57%) and Black race (38%). Parents reported increased child tooth brushing frequency during the pandemic. Parent interviews highlighted significant changes in family routines that impacted oral health behaviors and eating patterns, suggesting less optimal brushing and nutrition. This was linked to changed home routines and social presentability. Key informants described major disruptions in their oral health services and significant family fear and stress. In conclusion, the stay-at-home period of the COVID-19 pandemic was a time of extreme routine change and stress for families. Oral health interventions that target family routines and social presentability are important for families during times of extreme crisis.

## INTRODUCTION

The COVID-19 pandemic resulted in a disproportionately higher incidence of cases and deaths from COVID-19 in low-socioeconomic minority populations.^1,2 3^ The reasons for this are multi-factorial and not fully understood, but historical structural racism likely played a role.^4,5^ Many low-income Black and Hispanic communities experienced higher COVID-19 exposure rates related to occupation, transportation method, family structure, housing, and healthcare access. These factors are also known contributors to disparities in hypertension, obesity, diabetes mellitus, heart failure, and chronic obstructive pulmonary diseases, all of which increased the risk for worse outcomes with COVID-19.^6^

Another important marker of public health that had been well-described in the United States prior to COVID-19 is oral health.^7^ Oral health services and outcomes reflect social inequities and the limited integration of social, health, and dental services.^7^ Oral health risk factors were exacerbated by the pandemic. Stay-at-home orders impacted everyone, but the clinical dental field was impacted more than others. Dental clinics performed only emergency dental services.^8,9^ Dental practices started reviving services in June 2020, but the patient level was almost 64% less when compared to pre-COVID-19.^10^ Many people delayed treatment and preventive oral care.^11^

The purpose of this research is to contribute to our understanding of public health inequities by examining changes in oral health behaviors in low-income families in the Chicago region of the United States during the stay-at-home period of the COVID-19 pandemic. To do this, we followed participants in the Coordinated Oral Health Promotion (CO-OP) Chicago Trial (NCT03397589) during the first year of the COVID-19 pandemic. The objective was to identify future intervention targets regarding oral health behaviors, nutrition, and dental care access for low-income urban families with young children. Having previously observed a relationship between adult assistance with brushing and child oral health,^12^ we hypothesized families would report disrupted home oral health routines, food insecurity, distrust of dental services, and challenges accessing dental care. Understanding day-to-day behaviors of these families during a time of extreme crisis will help us design and support better services for families in the future.

## METHODS

### Design:

We used a mixed-methods approach (explanatory sequential design)^13^ including quantitative survey data from parents, in-depth parent interviews, and key informant community interviews with clinical and social service agencies associated with the families. Quantitative survey data guided interview questions with parents to allow for deeper exploration of issues. Interview data from key informants in clinical and social service agencies was intended to provide additional details on system operations and family experiences. The CO-OP Chicago Trial was designed as a cluster-randomized controlled trial, with one arm receiving intervention and the other a wait list control.^14^ The intervention had no impact on child brushing behavior or plaque score.^12^ Therefore, we combined trial participants into a single cohort.

### Eligibility/Recruitment:

#### Caregiver Surveys:

Survey eligibility criteria were: (1) being the caregiver of a child enrolled in the CO-OP Chicago Trial and still receiving services in Cook County, IL, and (2) gave permission on their initial consent to be contacted in the future for other studies (N = 395). Eligible families were mailed and/or emailed information on the study and then contacted by the research staff via telephone or email to assess interest and confirm eligibility.

#### Caregiver Interviews:

Following the caregiver survey, caregivers were asked if a future interview would be of interest. From those who said yes, we selected 21 caregivers to interview, based on reported race, ethnicity, and geographic location to ensure representativeness sample. Research staff called or emailed caregivers to verify their interest and schedule an interview.

#### Key Informant (KI) Community Interviews:

CO-OP Chicago Trial participants were initially recruited from ten medical clinics and ten special Supplemental Nutrition Program for Women, Infants, and Children (WIC) centers in the Chicago area.^9^ Inclusion criterion for these KI interviews was being a service provider or administrator in one of these 20 sites. Site contacts were emailed by the study team explaining the goals of the research and invited to recommend individuals at their sites for participation. These individuals were then contacted via email to verify interest and schedule interviews.

### Data Collection and Outcomes:

#### Caregiver Surveys:

The caregiver surveys were conducted by phone survey in English or Spanish. Survey questions were repeated from the trial (family demographics, brushing frequency, previous dental visit details, medical and dental insurance, oral health quality of life, water sources, sugary beverage and food consumption, social support, and psychosocial stressors).^14^ We added the USDA Food Insecurity Questionnaire^15^ and asked families their COVID-19 experiences and beliefs. Surveys took 25–117 minutes to complete. Caregivers were compensated $40 via gift card or check.

#### Family Interviews:

The project manager coordinated video conference (Zoom) times with caregivers, although many caregivers kept their video off due to technology issues or personal preference. Interviews, led by the principal investigator or project manager, lasted 37–73 minutes and were recorded and transcribed. Participants were compensated $100 via gift card or check. Interviews explored the following domains: COVID-19’s overall impact on the family; diet and nutrition changes; oral care; medical care changes for the family; dental care changes for the family; telehealth experiences; and how COVID-19 changed participants’ life and community.

#### KI Interviews:

The project manager coordinated a time for data collection via video platform. Interviews, led by the principal investigator, lasted 23–42 minutes and were recorded and transcribed. Participants were not offered compensation. Interviews explored COVID-19 impact on service delivery; impact on patients/clients; interventions to support patients/clients; and oral health.

### Analysis:

#### Caregiver Surveys:

Descriptive characteristics were reported using percentages or means with standard deviations, for categorical and continuous variables respectively. A multivariate logistic regression model was fit to the pandemic-era data, to predict child brushing frequency from covariates of interest. The final sample (n = 244) for regression analyses were participants with no missing values on these covariates. The model predicted child tooth-brushing frequency, dichotomized as high (i.e., twice a day or more) versus low (i.e., less than twice daily), with low being the reference. Significant covariates were selected from an initial set of variables including: original treatment group membership; study site type (clinic vs. WIC), site size, and most common racial/ethnic group at site; child gender, child age in months at final measurement time; caregiver age in years, caregiver highest educational degree; caregiver mouth condition, brushing frequency and overall health; child or caregiver health insurance status/source; child’s last dental visit, whether greater or fewer than 6 months; caregiver relationship status; child brushing assistance; caregiver functioning and emotional support; type of household drinking water source; toothpaste fluoridation; an index of household chaos; and frequencies of daily difficulties interfering with child’s oral care.

#### Family and KI Interviews:

Qualitative data were analyzed using a modified Grounded Theory approach.^16,17^ For the family interviews, three coders independently coded the first transcript using NVivo 1.6.1 (QSR International). Discrepancies were discussed, the initial codebook modified, and another three transcripts were triple coded and compared, with adjudication after each. The remaining 18 transcripts were single coded. The KI interviews followed a similar process using ATLAS.ti 7.5.16 Qualitative Data Analysis software (Atlas.ti. Scientific Software Development. Berlin, Germany) with one coder who reviewed coding decisions with the principal investigator. The second phase analysis consisted of conceptual coding. In a group process including the principal investigator, project manager, and coders, data were organized into final domains and themes.

### Human subjects:

Institutional Review Boards at the University of Illinois Chicago [2017 − 1090], and Chicago Department of Public Health [16 − 06] approved the study. Participants provided verbal informed consent.

## RESULTS

### Participant Descriptors (Table 1)

Surveys were conducted from 11/11/2020 to 05/17/2021. Of 395 eligible caregivers,^14^ 254 participated (64.3%). Most (98.8%) were female, with average age 30 (SD 6.5) years. Caregivers were mainly Hispanic ethnicity (56.7%) or Black race (39.8%), with 27.2% having a high school/GED degree or some college (57.5%). The current age of children at the time of survey ranged from 37–76 months (mean = 53.3, *SD* = 7.5), with 133 (52.4%) being female.

Thirty-two caregivers were offered interviews; 21 completed them (65.6%) between 06/17/2021 and 08/30/2021. Caregivers who completed interviews tended to have more education, and fewer were single. KI interviews (15 interviews from 25 total participants) were conducted from 12/3/2020–2/4/2021. All 20 sites were represented (13 participants from WIC sites and 12 from clinics). Twelve participants (48%) were medical providers and or administrators in clinics, 11 (44%) were WIC administrators, and 2 (8%) were WIC service providers. KIs were mostly female (88%); race/ethnicity was White (48%), Black (28%), other (24%), and Hispanic (20%).

Other data were captured to inform family economics. Food security was self-reported on the survey as marginally adequate for 27%, low for 24% and very low for 8%. Sixty-one percent said they received free school meals, and 35% used food banks. Many reported insufficient income to meet the family needs (21%) or just enough (44%). Forty-six percent reported recent household unemployment during COVID and 20% anticipated employment loss within the next 4 weeks. Mothers were doing most childcare (91%), with childcare often conflicting with work (37%). During a parent interview, one mother said: “*… from like 7:30 up until 10 o’clock at night sometimes they [her children] were with me at work outside in the car*.”

COVID-19 risk factors and beliefs were collected to determine sample generalizability. Just under half of survey participants (42.5%) had a household member at risk for COVID-19 due to essential employment or public transit reliance; 46.3% required quarantine at some point. Fifty-six (22.1%) reported a household member diagnosed with COVID-19. Most felt that the following actions were effective or very effective at keeping them safe: face masks (81.8%), hand washing/sanitizer (92.9%), avoiding public spaces (89.7%), avoiding contact with high-risk people (92.5%), and avoiding hospitals/clinics (79.8%). Survey participants reported doing a range of behaviors to keep safe (Table 2). During the family interviews, the majority agreed with masking and social distancing. They described challenges associated with these practices that included inability to see loved ones, offending family members, and feelings of physical and social isolation. Ten (48%) of family interview participants were unsure, scared, or disagreed with COVID-19 vaccination for both themselves and their children. One parent said *“I kind of feel like people who get it are like the guinea pigs of it to see if it works, to see how it - I’m going to wait because I feel like it’s safer for me to wait.”*

### Brushing and Nutrition Behaviors

Child brushing frequency per caregiver survey report increased during the trial and this trajectory continued during the pandemic (Table 3). Only 20 (7.9%) caregivers directly reported that brushing frequency changed during COVID-19. Two covariates were statistically significant in the logistic model for the data during COVID-19: caregiver’s brushing frequency (b=−1.60, p < .0001), and everyday life activities which made brushing difficult (p < .05). The strongest predictor was caregiver brushing frequency. Among the 43 caregivers who reported brushing their own teeth less than twice daily during the pandemic, 74% reported their children likewise brushed less than twice daily. For the 211 children whose caregivers reported brushing their own teeth at least twice daily, 89% reported their children also brushed at least twice daily. The household chaos measure was nonsignificant in the combined model but was significantly correlated with dichotomized brushing frequency (r=−.20, p < .005).

However, family interviews described a more complex pattern of child brushing during COVID-19. When brushing frequency was explored slowly in depth during interviews, almost half of parents (N = 10) said their children brushed less. This was linked to forgetting, but also to not worrying anymore about being socially presentable (Table 4). Some parents mentioned that day care had provided a backup for the home routine, and how loss of that translated into less brushing. Five said brushing frequency was unchanged; several parents made comments that tooth brushing had been and remained a low priority. Multiple parents attributed the responsibility of remembering to brush to the children.

Decreased frequency of consumption of sugary drinks and sugary food was reported on the survey relative to previous waves (Table 3), but parents and key informant in interviews reported more snacking (Table 4).

### Dental and Medical Care Access

The proportion of surveyed children to have visited the dentist in the past 6 months was less during the COVID-19 survey (61.1% declined to 56%), as shown in Table 3. Thirty-seven percent (N = 93) reported missing dental appointments for any family members since COVID-19 started. The most common reasons for missing appointments were to avoid being around others (25.8%), because the clinic was closed (21.5%), to avoid healthcare settings (18.3%), and because the clinic cancelled the appointment (18.3%). Caregivers generally agreed with clinical safety restrictions (Table 5). Caregivers reported an increase in the amount of index children with caries, from 13.7% at the trial end to 23.7% during COVID-19. When asked if anyone in the household had needed dental care during COVID-19 but could not get it, 24% said yes. The main reason was because of problems getting in due to COVID-19 (55.7%), followed by insurance coverage (13.1%).

This was confirmed by the interviews, where clinics and parents described cancelled and postponed care. They described in detail how clinical safety restrictions–especially only one child/parent per visit–caused real challenges and limited how families accessed care. Key informants described perceiving fear and mistrust from their patients about seeking medical care (Table 4). Most key informants said oral health and dental care access was a low priority for them for their patients during COVID. In the family interviews, mothers reported most of the cancelled care was quickly resolved.

## DISCUSSION

This is one of the first studies to describe the impact of the stay-at-home portion of the COVID-19 pandemic on oral health behaviors among urban families with very young children. Our results reinforce that oral health behaviors and appearance are closely linked^18,19^ and suggest interventions focus more on social presentability as a driver for toothbrushing. We also found that the importance of caregivers as role models and facilitators of their children’s oral health behaviors^19^ remains important during times of crisis. These results will help in program development aimed at supporting families during times of crisis.

The proportion of children in the cohort with adequate daily toothbrushing reported on the survey was higher during the pandemic than expected. Conceivably, family changes during the pandemic improved hygiene (e.g., additional time at home, less rushed morning schedules, increased concerns about cleanliness). This was reported in by Ciardo et al., in a study conducted with adults in Germany.^20^ In our survey cohort, as in prior research,^12,19^ caregiver brushing appeared to be the most powerful predictor of child brushing frequency during the pandemic. This strong relationship may be due to direction or modeling of adult to child, or to shared factors affecting both. Response bias (such as social desirability or biased recall) for both variables may have occurred. However, the most likely explanation for the increase in child brushing frequency reported by our cohort during the pandemic is the maturation of the children. The family interviews add to these results by describing more details of the complicated brushing picture and the challenge of capturing a complex behavior with a single question in a survey; some parents who initially said brushing happened regularly during COVID changed their responses when interviewers probed more about routines.

Our findings emphasize previously described social and programmatic structures that support consistent brushing behaviors. While it is well known that daily routines are critical for maintaining behaviors such as brushing,^19,21^ our results highlight the importance of social presentability and accountability as motivators for establishing those routines. During interviews, parents explained that when children did not have to be anywhere, brushing routines changed. Families could have established new brushing routines in the stay-at-home environment but almost said they did not, often because of a reduction in their perceived importance of brushing when no one was going to leave the house. Our data also describe the role that formal childcare providers play in supporting oral health behaviors.^22,23^ This is especially important when families place independent brushing responsibility on children too young to adequately manage the task alone.

Similar to brushing frequency, survey results for snack and sugary drink consumption did not align exactly with family interviews. The survey sample reported less consumption over time but parents described more consumption of sugary snacks and drinks during COVID-19 in interviews. In family interviews, parents mentioned they did more cooking and had more family eating time, but they also described more opportunities for children to obtain snacks and overall boredom that led to eating. Gotler et al.,^24^ and Campagnaro et al.,^25^ also reported significant increases in child snacking during the pandemic. This may be due to increased access to food throughout the day, and potentially contributed to worse oral health.

Although participants reported more cancellations of scheduled dental appointments by dental clinics and limited dental care access during the COVID-19 pandemic, few participants reported falling behind on clinical dental care. While others have reported significant delays of dental services,^20,25^ perhaps the impact over time was less severe than initially feared. It is also possible that the Chicago area was able to resume dental services more quickly than other areas. Participants described a range of precautions in place and generally agreed with these precautions. It is possible that awareness of the recommended schedule for and importance of clinical dental care for prevention in our population was low, leading parents to not recognize limitations in access. We also wondered if delaying dental services was one of the few risks that parents felt they could control; they could not stop going to work or stores, but they could avoid clinical settings.

This study has numerous limitations. The children in our sample began at approximately the same age and experienced normal age-related changes in behaviors that made it impossible to differentiate COVID-19 effects from normal maturation, or from factors related to participation in the study itself. Survey and interview data reported via the telephone are subject to respondent, interviewer, and recall biases. The differences in results raised by our mixed methods analysis shows the limitations of closed-ended survey questions. Perhaps one of the most significant challenges in interpreting these results was the constantly changing nature of the COVID-19 pandemic. Many questions asked about specific time periods, such as the prior 7 days or the last month. Situations were changing day by day, and the responses we recorded may not have been able to properly capture the entire trajectory of experiences. Finally, generalizability is limited because this research was conducted in a single urban low-income geographic area.

### Conclusions

This mixed methods analysis highlights the complexity involved in child tooth brushing and other oral health behaviors associated with caries risk. While our survey data reported increased brushing frequency during the pandemic, our qualitative data emphasized that routines, school and daycare programmatic support (food provision and toothbrushing support), and social presentability contributed greatly to actual brushing. COVID-19 may have caused a temporary change in tooth brushing habits and dietary patterns for many families, increasing their risk for worse oral health outcomes. This research was conducted in a population with significant economic fragility and high COVID-19 risk early in the pandemic. Our results highlight areas where oral health interventions can be targeted in the future to better support families.

## Figures and Tables

**Table 1:** Participant Demographics

	Caregiver Survey N=254	Family Interviews N=21	Key Informant Interviews N=25
Caregiver Female (%)	251 (98.8%)	21 (100%)	22 (88%)
Caregiver Age (years), mean (SD)	30 (6.5)	32 (6.3)	NA
Caregiver Race (%)			
Black	101 (39.8%)	8 (38.1%)	7 (28%)
White	31 (12.2%)	2 (9.5%)	12 (48%)
Other	102 (40.2%)	10 (47.6%)	6 (24%)
Caregiver Hispanic (%)	144 (56.7%)	13 (61.9%)	5 (20%)
Caregiver Highest degree earned (%)			
Less than high school	39 (15.4%)	2 (9.5%)	NA
High school/GED	69 (27.2%)	3 (14.3%)	
More than high school	146 (57.5%)	16 (76.2%)	
Caregiver Relationship status (%)			
Single	96 (37.8%)	6 (28.6%)	NA
Living with partner/spouse	158 (62.2%)	15 (71.4%)	
Child Female (%)	133 (52.4%)	13 (61.9%)	NA
Child’s age at time of COVID-19 survey (months), mean (SD)	53.3 (7.5)	55.1 (5.9)	NA
Household size, mean (SD)	2.0 (.9)	2.0 (.7)	NA
Children in household, mean (SD)	2.5 (1.2)	2.5 (1.4)	NA
Child has health insurance (%)	251 (98.8%)	20 (95.2%)	NA
Child health insurance source (%)			
Medicaid	225 (89.6%)	19 (95.0%)	NA
Other	26 (10.4%)	1 (5.0%)	
Child has dental insurance (%)	237 (93.3%)	19 (90.5%)	NA
Child dental insurance source (%)			
Medicaid	208 (81.9%)	18 (85.7%)	NA
Other	46 (18.1%)	3 (14.3%)	
Professional role (%)			
Medical provider/administrator	NA	NA	12 (48%)
WIC administrator			11 (44%)
WIC service provider			2 (8%)

**Table 2:** Things families have done to keep safe from COVID-19 (N=254)

Washed/sanitized hands	249 (98.8%)
Worn a face mask	247 (98.0%)
Avoided in-person contact with high-risk people	246 (97.6%)
Avoided social gatherings	241 (95.6%)
Avoided public places/crowds	233 (92.5%)
Avoided in-person contact with friends/family	220 (87.3%)
Stockpiled food or water	198 (78.6%)
Cancelled/postponed travel	188 (74.6%)
Cancelled/postponed work or school activities	166 (65.9%)
Worked/studied from home	152 (60.3%)
Visited dental/health care provider in person	147 (58.3%)
Cancelled dental or health care appointment	115 (45.6%)
Isolated from others who live with me	84 (33.3%)

**Table 3:** Brushing Behaviors, Dental Care Utilization, and Other Health Factors (N=254)

	Baseline	6-Month	12-Month	During COVID-19	*Caregiver said changed from prior to C0VID-19*
**Brushing Behaviors**					
Child brushing 2x/d or more	48.4%	57.4%	61.6%	78.4%	*7.9%*
Brushing duration 120 sec or more	32.5%	29.6%	32.6%	51.4%	*6.3%*
Fluoride toothpaste	24.0%	48.4%	65.3%	84.3%	*5.6%*
Correct amount of toothpaste	56.7%	53.7%	49.6%	20.9%	*5.5%*
Caregiver always helps child brush	58.3%	47.1%	41.3%	37.8%	*8.3%*
Family/partner help with brushing (always or sometimes)	92.5%	93.9%	89.3%	87.4%	*3.5%*
ADLs interfere with brushing					*4.3%*
Never/rarely	70.1%	75.0%	77.7%	79.9%	
Sometimes	23.6%	15.2%	15.7%	13.0%	
Most/all the time	6.3%	9.8%	6.6%	7.1%	
Caregiver brushing 2x/d or more	76.0%	77.5%	78.9%	83.1%	*3.5%*
**Dental Care Utilization**					
Child saw dentist in last 6 months	34.4%	48.4%	61.1%	56.0%	*NA*
Caregiver last visited dentist					*NA*
<1 yr	56.5%	58.0%	61.4%	72.9%	
1–2 yrs	22.5%	17.7%	19.1%	27.1%	
>2yrs	21.0%	24.3%	19.5%	0%	
**Health, Quality of Life, and Nutrition**					
Index child has caries	7 (2.8%)	18 (7.4%)	33 (13.7%)	59 (23.7%)	*NA*
Child oral health QOL (ECOHIS), mean (SD)	2.4 (3.0)	1.9 (2.9)	1.6 (2.6)	1.6 (3.9)	*NA*
Caregiver general health					*5.1%*
Fair/Poor	22.4%	25.8%	19.4%	25.6%	
Good	40.2%	37.3%	47.1%	47.6%	
Excellent/Very good	37.4%	36.9%	33.5%	26.8%	
Caregiver condition of mouth/teeth					*NA*
Very good/Good	42.1%	45.9%	47.9%	52.8%	
Fair	46.5%	43.9%	40.5%	35.4%	
Poor	11.4%	10.3%	11.6%	11.8%	
Caregiver oral health QOL (OHIP), mean (SD)	6.8 (8.2)	6.5 (7.8)	6.2 (8.0)	7.7 (10.3)	*NA*
Sugar sweetened beverages (>1/day)	37.8%	45.5%	39.4%	35.8%	*14%*
Sugary foods (>1/day)	31.5%	32.4%	31.0%	22.8%	*13%*

**Table 4:** Selected Parent, Clinic Provider, and WIC Administrator Interview Quotes

Domain	Interview Quotes
Social presentability and toothbrushing, forgetting	…*”I think once staying home and kind of, I don’t know, you kind of get lazy and you just didn’t really think about it.”* [parent]
	*“Yeah, it’s like, well, you got nowhere to go*,… *I’ll just do it later and just forgot and that sort of stuff.”* [parent]
Attributing brushing responsibility to young children, forgetting	*“My focus kind of drifted, but they old enough to know to brush their teeth but my focus honestly it did kind of drifted to basically getting them to school on time, making sure they’re in front of the camera so they don’t get marked absent*…*”* [parent]
Nutrition, frequency of eating	*“I think a lot of people ate more during Covid, especially my fears that don’t let me go out. I gained a lot of weight over the course of this Covid because you’re in the house. You’re eating more and you know like you’re doing the e-learning only* [parent]
	*“My refrigerator was always open, especially with my 15-year-old. He thought it was the open cafe for him ever since he wakes up.”* [parent]
	*“Yeah, lots of snacking going on and everybody attributes it to the pandemic. Well, we’ve been in the house. We’re eating more of this”* [clinic provider]
Fear and mistrust of medical care	*“The other thing* I *think it has changed is* I *feel they’re like more fearful so they don’t even want to put a foot in the clinic, so even having to reach those patients, like please come back, has been challenging as well.”* [clinic provider]

**Table 5:** Parent beliefs regarding COVID-19 Clinical Protocols (N=252)

Believe it is a good rule:	
Temperature screening.	245 (97,2%)
Masks required.	245 (97.2%)
Illness screening questions.	243 (96.4%)
Waiting in cars or outside to keep the number of people in the waiting room down.	229 (90.9%)
Only one parent allowed with child.	228 (90.5%)
Bringing only one child to appointments.	212 (84.1%)
COVID-19 testing before visit	186 (73.8%)
Parent does not go with child into parts of the clinic.	80 (31.8%)
